# Comparative Analysis of Foot Temperature Associated with the Use of Sports and School Footwear in Children Aged 9 and 10 Years

**DOI:** 10.3390/children13101436

**Published:** 2026-10-19

**Authors:** Marina Fontán-Jiménez, Raquel Sánchez-Rodríguez, María Victoria Cáceres-Madrid, Alfonso Martínez-Nova, Beatriz Gómez-Martín, Elena Escamilla-Martínez

**Affiliations:** Nursing Department, University of Extremadura, 10600 Plasencia, Spain; marinaf@unex.es (M.F.-J.); pgvicky@unex.es (M.V.C.-M.); podoalf@unex.es (A.M.-N.); bgm@unex.es (B.G.-M.); escaelen@unex.es (E.E.-M.)

**Keywords:** thermography, foot, child, footwear, skin temperature

## Abstract

**Highlights:**

**What are the main findings?**

- After three hours at school, sports footwear was linked to ~1 °C warmer feet than leather school footwear on the medial and lateral top of the foot.
- Smaller differences on the sole and toes were not significant after correction but may deserve further study.

**What are the implications of the main findings?**

- Both footwear types kept foot temperature within the usual comfort range, but effects on comfort or skin health remain unknown.
- Thermal properties should be considered when choosing school footwear; the closer fit of sports shoes may contribute to these higher temperatures.

**Abstract:**

**Background/Objectives:** Children’s footwear plays a key role in foot protection and development, influencing both mechanical and thermal comfort. However, there is limited evidence on how different types of footwear affect foot thermoregulation in the pediatric population. Therefore, the aim of the present study was to analyze the thermal pattern of the pediatric foot and to compare temperature differences in plantar and dorsal regions following the use of school and sports footwear. **Methods:** A total of 108 schoolchildren (61 girls and 47 boys), with a mean age of 9.46 ± 0.52 years, participated in the study. A non-randomized within-participant repeated-measures crossover design was used, in which each participant was assessed wearing both types of footwear on two separate days, with each child therefore serving as their own control. Thermographic measurements were performed using a Flir^®^ E60bx camera (FLIR Systems, Wilsonville, OR, USA) after 3 h of school activity. Footwear conditions were compared within each region of interest (ROI) of the plantar and dorsal surfaces using the Wilcoxon signed-rank test, with Benjamini–Hochberg false discovery rate (FDR) correction. **Results:** Regardless of the footwear used, the highest temperatures were recorded in the medial longitudinal arch on the plantar surface and in the lateral region on the dorsal surface, whereas the lowest temperatures were observed in the fifth toe on both surfaces. Sports footwear was associated with higher mean temperatures than school footwear in all ROIs (0.61 to 0.95 °C). After FDR correction, significant differences remained only in the medial and lateral dorsal regions (+0.95 and +0.94 °C, respectively; q = 0.0139), whereas none of the plantar differences remained significant. A strong period effect was also observed, with foot temperatures 1.28 to 2.24 °C higher at the second visit regardless of footwear; after adjusting for this effect, temperatures remained higher with sports footwear in all ROIs (0.71 to 1.04 °C). **Conclusions:** The pediatric foot exhibited a consistent thermal pattern across footwear conditions. However, sports footwear was associated with an approximately 1 °C higher temperature in the medial and lateral regions of the dorsal foot, an effect that persisted after accounting for the period effect. These findings underscore the importance of thermoregulatory properties in children’s footwear selection.

## 1. Introduction

Children’s footwear plays an important role in the healthy development of the foot, acting during this stage of structural maturation as a protective barrier against the environment and facilitating both mechanical and thermal comfort for the wearer [1]. From this perspective, footwear design should address ergonomic criteria, prioritizing comfort, adaptability and breathability [2]. Children’s footwear should be durable and flexible, with a straight last that allows proper foot alignment and adequate toe box space to prevent digital compression [3,4]. The materials used in footwear manufacturing are directly related to the dissipation of heat generated by the foot and to perceived thermal comfort. Although breathability and thermal properties depend not only on the origin of the material (natural vs. synthetic) but also on upper construction, thickness, perforation, lining, membrane properties, and shoe design, natural leather generally provides greater durability and breathability, in addition to adapting to potential foot deformities without exerting pressure, whereas some modern synthetic materials exhibit good sweat absorption and breathability properties, although their durability is generally lower [5].

From a physiological perspective, maintaining foot temperature within an optimal range, estimated between 20.00 and 33.00 °C, is essential to ensure thermal comfort [6]; however, this temperature range was established for the in-shoe microclimate of protective footwear in adults, and its applicability to the pediatric population remains uncertain. When temperature deviates from these values, thermoregulatory responses such as cutaneous vasoconstriction or sweating are triggered [6], which, in our view, may compromise the child’s overall well-being. Thermal stability may also be an important factor in concentration and physical performance [7].

To accurately and safely measure foot surface temperature, infrared thermography represents an effective, non-invasive, painless and contactless technique that does not emit ionizing radiation and has no known side effects [8]. However, despite its utility, to our knowledge, few studies have directly related foot temperature to the type of footwear used, particularly in the pediatric population. In our view, analyzing temperature could help to identify alterations in heat dissipation and thermoregulatory mechanisms associated with different types of footwear, as well as temperature variations linked to mechanical friction, localized pressure, insufficient ventilation or functional changes in superficial microcirculation.

Prolonged elevated foot temperatures, in combination with friction and prolonged moisture exposure, have been associated with the development of blisters, friction dermatitis and fungal infections [3,6,9,10]. We consider that this may be particularly relevant in children, although direct evidence on the vulnerability of the pediatric foot to heat, moisture accumulation and friction within footwear is limited. Conversely, an excessively cold environment may lead to cutaneous vasoconstriction, reduced superficial perfusion and increased susceptibility to irritation and micro-tissue lesions [11]. Therefore, the study of foot temperature may contribute to optimizing the design and selection of children’s footwear from a preventive perspective, aimed at preserving foot health, well-being and functional development during critical stages of growth. We hypothesized that sports footwear would be associated with higher foot temperatures than school footwear and that these differences would vary across plantar and dorsal regions.

The primary aim of the present study was to analyze the thermal pattern of the pediatric foot and regional temperature differences (plantar and dorsal surfaces) following the use of two types of footwear, school and sports, commonly worn by this population. This information will enable the identification of regional foot-temperature differences, providing relevant data for both manufacturers and child health professionals, as well as for parents, who are responsible for selecting the most appropriate footwear for their children.

## 2. Materials and Methods

### 2.1. Participants

This study used a non-randomized within-participant repeated-measures crossover design. The study was conducted in two state-funded primary education schools in Mérida (Badajoz, Spain). Data were collected during the last three weeks of March 2018. A convenience sample was used, and no a priori sample-size calculation was performed. A total of 162 schoolchildren were invited to participate. The following inclusion criteria were applied: being aged between 9 and 10 years and presenting no apparent structural foot deformities. Participants were excluded if they (a) had fever or malaise at the time of data collection; (b) had any systemic disease; (c) had previously applied topical products to the foot, such as creams or ointments, or were undergoing treatment for a cutaneous lesion; (d) had performed physical exercise prior to data collection [12]; or (e) were undergoing orthopedic treatment. Of these 162 children, 54 were excluded because they met one or more of the exclusion criteria, because they or their parents were unwilling to participate, or because they were absent from school on one or more of the days scheduled for data collection. The final sample comprised 108 participants (61 girls and 47 boys), with a mean age of 9.46 ± 0.52 years, a mean height of 135.79 ± 6.50 cm, a mean body mass of 33.82 ± 7.23 kg, and a mean body mass index (BMI) of 18.19 ± 2.80 kg/m^2^. As the participants were minors, their legal guardians provided written informed consent for their participation after being fully informed about the nature and procedures of the study. In addition, the study was approved by the Bioethics and Biosafety Committee of the University of Extremadura (ID: 28/2018).

### 2.2. Procedure

**Figure 1:**
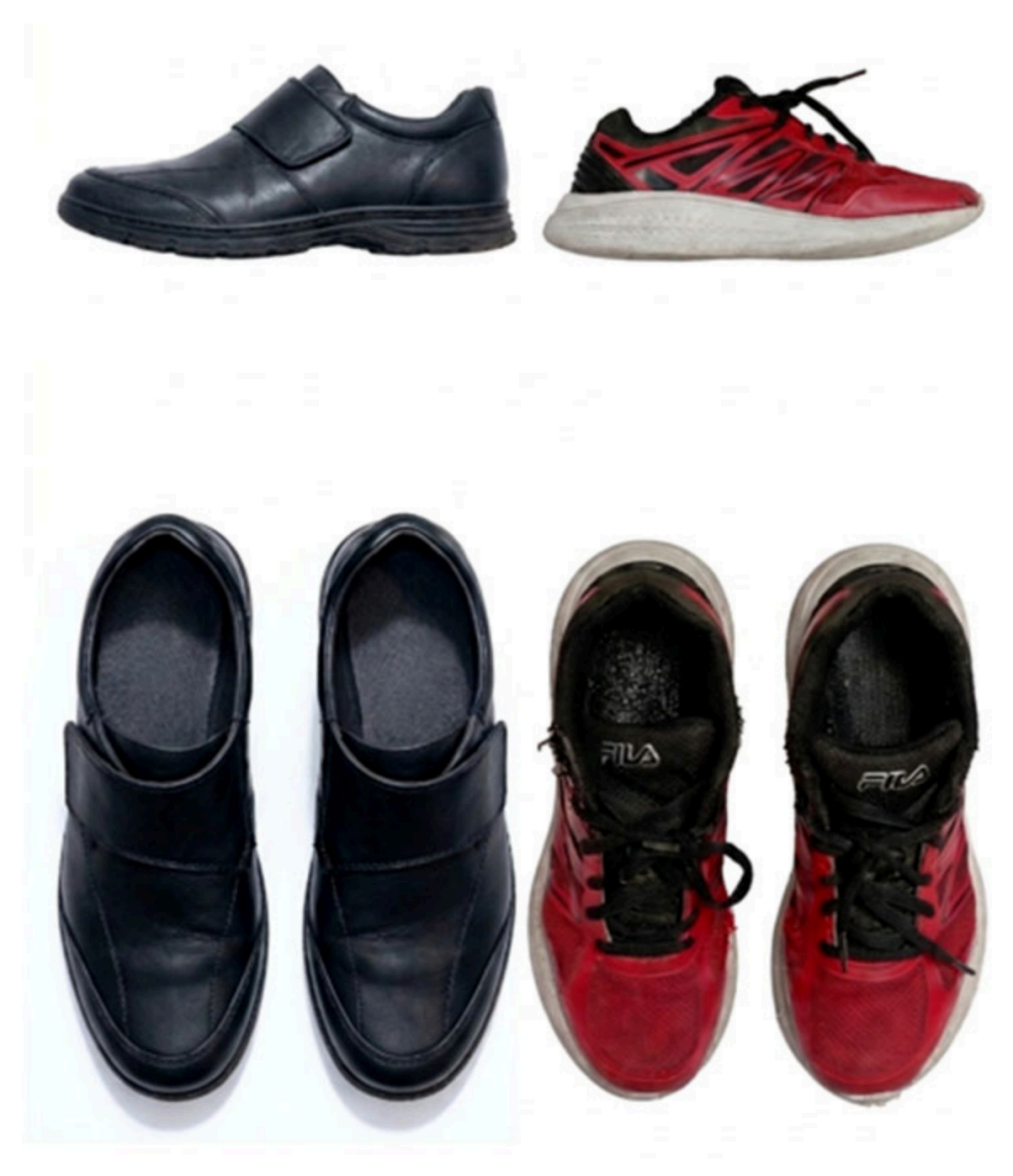
School Footwear (**left**) and Sports Footwear (**right**).

A clinician with more than 25 years of experience in the management of foot pathology collected data relating to biological and anthropometric variables (age, sex and BMI), selected participants according to the inclusion criteria, obtained all the necessary data for the study, and classified the children’s footwear as either school footwear or sports footwear (Figure 1). For each footwear category, footwear was accepted if it met a set of predefined general characteristics, and all models formed part of the official school uniform of the participating schools. School footwear was required to feature a natural leather upper (1.2 to 1.6 mm thickness), a natural leather or cotton textile lining, Velcro^®^, lace or buckle fastening [13], a polyurethane midsole (10 to 20 mm thickness), a thin and flexible sole (6 to 10 mm) made of thermoplastic rubber or natural non-slip rubber [14], and a removable insole of leather or natural fabric, with no additional cushioning elements. Sports footwear was required to have a synthetic mesh upper (polyester or nylon) with thermoplastic polyurethane reinforcements, a synthetic lining, lace or Velcro^®^ fastening, an ethylene-vinyl acetate (EVA) midsole (15 to 25 mm thickness) providing cushioning, a rubber outsole [1], and a total sole stack height of 20 to 35 mm.

#### 2.2.1. Allocation

Data collection was conducted with an interval of 8 to 9 days between assessments. The type of footwear assessed during the first visit depended on each child’s usual school routine and the footwear they were scheduled to wear on that day. During the first assessment, 57 children were evaluated wearing sports footwear and 51 wearing school footwear. At the second assessment, each participant was evaluated wearing the alternative type of footwear.

#### 2.2.2. Thermographic Analysis of the Feet

Thermographic measurements were performed after the first three hours of the school day, immediately before the scheduled recess period. During these three hours, although physical activity was not objectively recorded, children followed their usual classroom activities, which mainly involved sitting during lessons, standing and walking short distances within the classroom or school facilities, and other routine low-intensity school activities. Physical education classes were not included during the assessment period. Children were instructed to avoid running, jumping, vigorous physical activity, or other activities that could substantially increase body temperature or peripheral blood flow. Measurements were performed before recess to avoid the potential influence of more intense physical activity during the break. The same procedure was followed at both assessment visits. All children wore the same brand of socks, as required by the school’s uniform policy.

In each school, the study was conducted in a spacious room, free from air currents, with uniform artificial lighting and without nearby electronic equipment that could interfere with the measurements [12]. In addition, room temperature and relative humidity (RH) were monitored using a Flir^®^ MR77 thermo-hygrometer (FLIR Systems, Wilsonville, OR, USA; air temperature accuracy ±2 °C and RH accuracy ±2.5% between 10% and 90% RH, according to the manufacturer; readings displayed with a resolution of 0.1 °C and 0.1% RH), recording values of 23.30 ± 1.14 °C and 60.25 ± 5.32%, respectively. Room temperature did not differ between the school-footwear and sports-footwear sessions (23.33 ± 1.24 °C vs. 23.27 ± 1.02 °C; Wilcoxon *p* = 0.5451), whereas RH was slightly lower in the sports-footwear sessions (59.10 ± 5.51% vs. 61.39 ± 4.88%; *p* = 0.0034). By visit, room temperature was higher and RH lower at the second visit than at the first (23.94 ± 1.08 °C vs. 22.67 ± 0.79 °C, and 57.90 ± 5.95% vs. 62.59 ± 3.21%, respectively; Wilcoxon *p* < 0.0001 in both cases), although all sessions were conducted during the same season.

**Figure 2:**
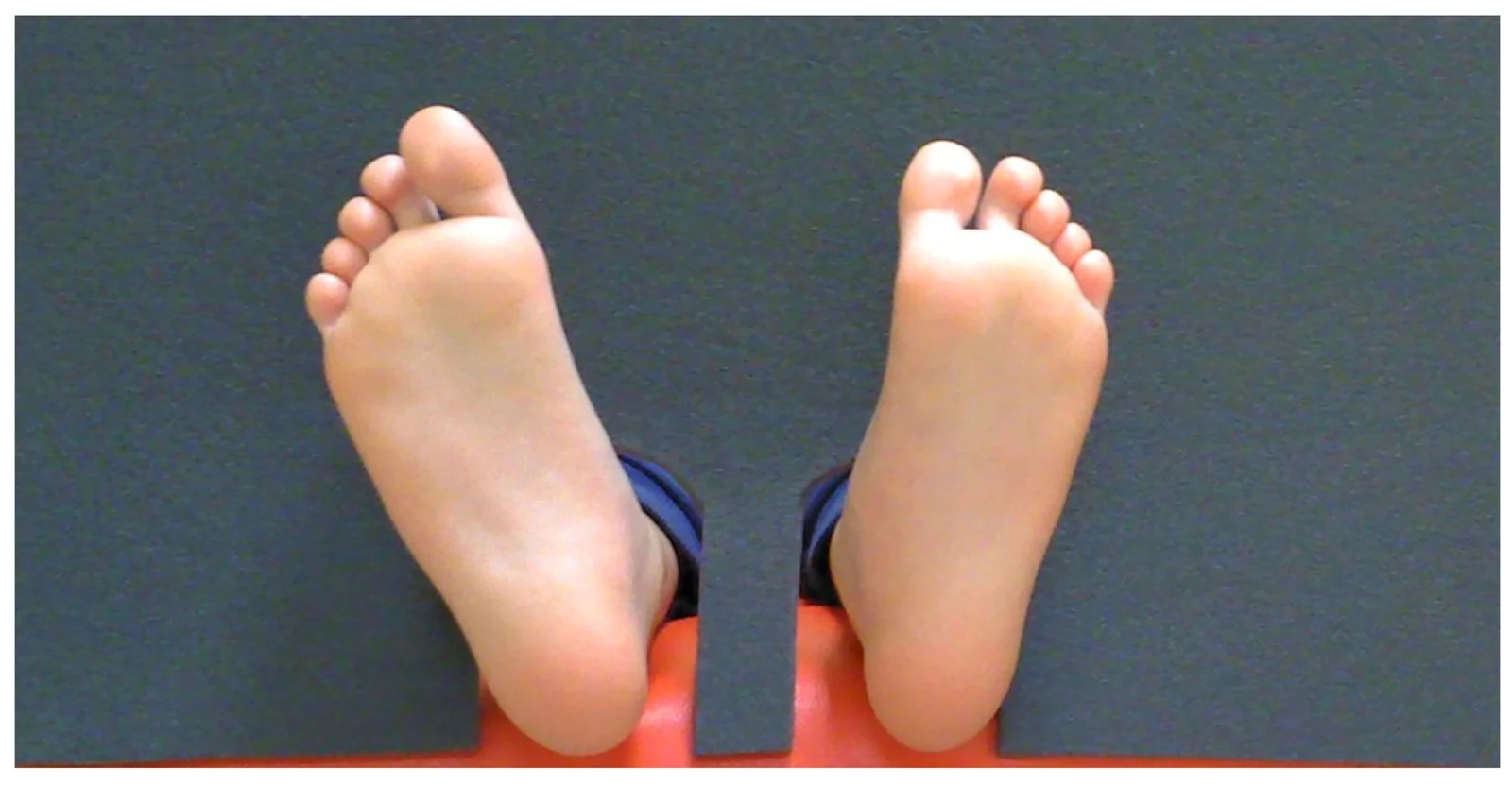
Black isolation screen.

For thermographic image acquisition, participants entered the examination room and were seated on an examination couch with their legs extended, maintaining the ankles at 90°. They were then instructed to hold a black screen at ankle level to isolate the feet from the rest of the body (Figure 2), thereby preventing body heat from being reflected in the thermographic image [12,15].

**Figure 3:**
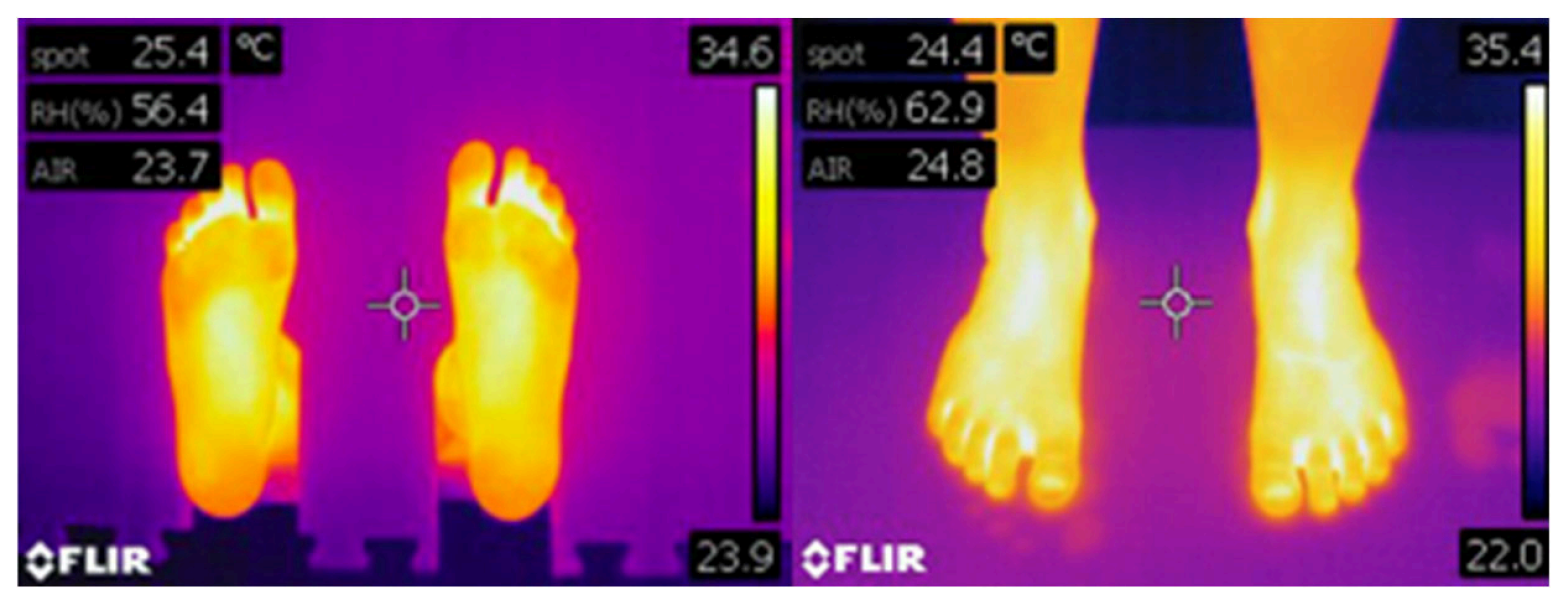
Thermographic image of the plantar surface of the foot (**left**) and the dorsal surface (**right**).

Subsequently, the examiner removed the footwear and socks, avoiding contact with the feet so as not to interfere with their temperature. Three thermographic images of the plantar surface were then immediately acquired (Figure 3). The camera used was a Flir^®^ E60bx thermal camera (FLIR Systems, Wilsonville, OR, USA), positioned on a tripod at 1 m from the participant. This camera has the following specifications: (1) a pixel resolution of 76,800 (320 × 240 pixels); (2) thermal sensitivity of 0.045 °C at +30 °C; (3) a temperature range from −20 °C to +120 °C with an accuracy of ±2 °C or ±2%; (4) a spectral range of 7.5 to 13 μm; and (5) an image frequency of 60 Hz. Images were acquired with an emissivity setting of 0.98 and the ironbow color palette. This camera model has previously been evaluated in a peer-reviewed study of skin temperature responses to exercise, in which it was compared with other infrared camera models and evaluator reproducibility was assessed [16], and has been used for plantar thermography in footwear research [17]. As both footwear conditions were assessed with the same camera, settings, distance and protocol, the paired comparisons depend mainly on the precision and repeatability of the camera rather than on its absolute accuracy.

The first plantar thermographic image was acquired within approximately 30 to 45 s of removing the shoes and socks. The second and third plantar images were obtained at 15 s intervals, allowing the camera to be refocused before each image. Thus, the three plantar images required approximately 60 to 75 s in total.

For acquisition of the dorsal foot image, the participant stood up from the examination couch and stepped onto a black mat made of EVA foam, 1 cm thick and measuring 60 × 60 cm. Subsequently, three images of the dorsal surface were captured with the camera positioned at 1 m distance, following the same procedure and requiring a similar time (approximately 60 to 75 s). Overall, the complete thermographic acquisition took approximately 2 to 2.5 min from footwear removal to completion of the six images.

After an interval of 8 to 9 days from the initial visit, a second thermographic assessment was conducted, this time using the alternative type of footwear (school footwear for those who had worn sports footwear during the first visit, and vice versa). The same previously described protocol was followed for image acquisition.

#### 2.2.3. Thermographic Image Processing

The images were processed using FLIR Tools+ software (version 6.4; FLIR Systems, Wilsonville, OR, USA). Different regions of interest (ROIs) were defined and located over anatomical areas of the foot, as this procedure provides good inter- and intra-rater reliability [18]. ROI placement and the extraction of the temperature values of each ROI in the three images were performed by the fourth author (A. M.-N.). These values were then provided to an external professional statistician, who calculated the mean of the three images for each ROI and performed the initial statistical analysis as a contracted service (see Acknowledgments). This reference supports the repeatability of ROI placement; the reliability of the temperature values was not assessed in the present sample, because only the mean of the three images per ROI was retained in the dataset available for analysis (see Section 4, Discussion).

**Figure 4:**
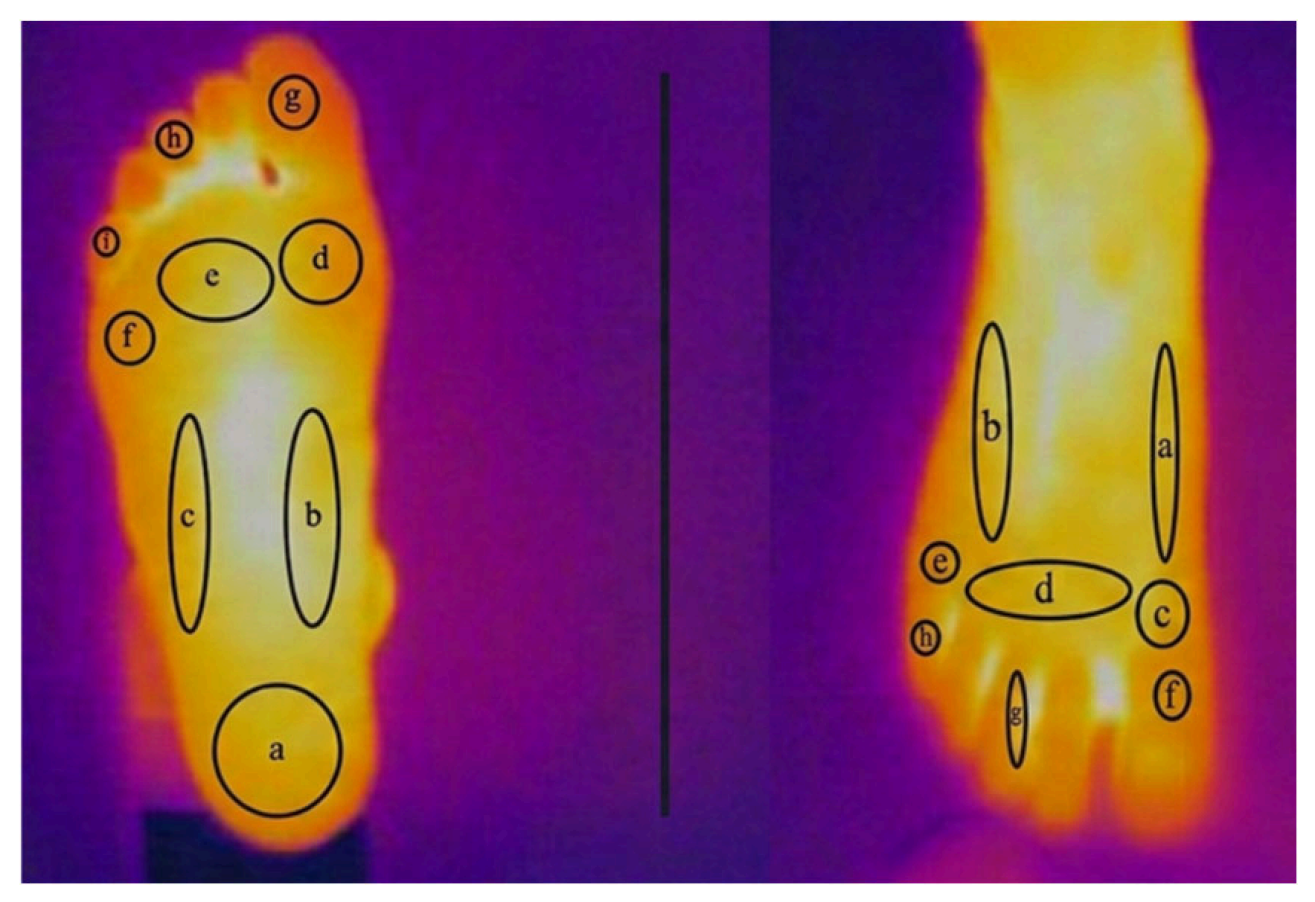
ROIs shown in the thermal image of the plantar surface of the foot (**left**) and the dorsal surface (**right**).

In the plantar images, the ROIs were (a) heel, (b) medial longitudinal arch (MLA), (c) lateral longitudinal arch (LLA), (d) first metatarsal head (1st MTH), (e) central metatarsal heads (central MTHs), (f) fifth metatarsal head (5th MTH), (g) first toe, (h) third toe, and (i) fifth toe (Figure 4). In the dorsal images, excluding the nail due to its different emissivity compared with skin [19,20], the following regions were defined: (a) medial region, (b) lateral region, (c) 1st MTH, (d) central MTHs, (e) 5th MTH, (f) proximal region of the first toe, (g) proximal region of the third toe, and (h) proximal region of the fifth toe (Figure 4). The researcher who performed the thermographic image processing (ROI placement and extraction of temperature values; fourth author, A. M.-N.) was blinded to the footwear condition.

### 2.3. Statistical Analysis

To maintain data independence [21], only temperatures from the right feet of the participants were considered, as stable and reproducible symmetry of temperature in homologous body regions has been demonstrated in healthy individuals [22,23,24,25]. The mean temperature for each ROI on the plantar and dorsal surfaces of the foot was calculated as the average of the three thermographic images obtained for each region.

Mean values, standard deviation, median and interquartile range (IQR) were computed for each footwear condition. The Kolmogorov–Smirnov test with Lilliefors correction was applied to assess the normality of the data distribution, both for each footwear condition and for the paired differences (sports minus school footwear). Temperatures with school footwear deviated from normality in all ROIs (*p* < 0.05) and those with sports footwear in five of the 17 ROIs (1st MTH, central MTHs and first toe on the plantar surface, and medial and lateral dorsal regions; *p* = 0.007 to 0.042), whereas the paired differences deviated from normality only in the medial dorsal region (*p* = 0.047). Given the non-normal distribution of the original variables and the presence of outlying values, the non-parametric Wilcoxon signed-rank test was retained for all paired comparisons as a conservative approach. The mean paired difference (sports minus school footwear) is reported with its parametric (t-based) 95% confidence interval (CI) for descriptive purposes only, because inference was based on rank-based tests; these intervals are not adjusted for multiplicity.

The Wilcoxon signed-rank test was applied to compare two related groups, and effect size was assessed using Rosenthal’s r. To account for multiple comparisons across the 17 ROIs, *p*-values were adjusted using the Benjamini–Hochberg false discovery rate (FDR) procedure; adjusted *p*-values are reported as q values. Statistical significance was defined as an FDR-adjusted q < 0.05; unadjusted *p*-values below 0.05 are described as nominally significant. Exact *p* and q values are reported to four decimal places. Because the order of conditions was not randomized, a period (order) effect was tested by comparing the within-participant differences (sports minus school footwear) between the two sequence groups (sports footwear first, *n* = 57; school footwear first, *n* = 51) with the Mann–Whitney U test, following the standard approach for two-period crossover designs [26]. As a sensitivity analysis, the footwear effect was re-estimated adjusting for period, by comparing the period differences (second minus first visit) between the sequence groups with the Mann–Whitney U test, with FDR correction across the 17 ROIs. Footwear and period effects were estimated as half the sum and half the difference, respectively, of the mean within-participant differences in the two sequence groups. A post hoc sensitivity analysis (paired design, *n* = 108, 80% power, noncentral t distribution) showed a minimum detectable effect of dz = 0.27 at a two-sided alpha of 0.05, rising to approximately dz = 0.37 at the Bonferroni-corrected alpha for 17 tests (0.05/17 = 0.0029). Statistical analysis was performed using IBM SPSS Statistics, version 22 (IBM Corp., Armonk, NY, USA).

## 3. Results

On the plantar surface (Table 1), following the use of school footwear, the highest temperature was recorded in the MLA, with a value of 30.64 ± 2.11 °C, whereas the lowest temperature was observed in the fifth toe, at 27.86 ± 2.90 °C. With the use of sports footwear, the highest temperature was likewise found in the MLA, with a mean value of 31.30 ± 1.27 °C. The lowest temperature, as with school footwear, was recorded in the fifth toe, with a mean of 28.47 ± 2.03 °C. On the dorsal aspect of the foot (Table 2), following the use of school footwear, the highest temperature was recorded in the lateral region, with a value of 31.15 ± 2.25 °C, whereas the lowest temperature was observed in the fifth toe, with a mean of 28.51 ± 2.95 °C. With sports footwear, the lateral region of the foot also showed the highest dorsal temperature, with a mean value of 32.09 ± 1.39 °C. The lowest temperature was again recorded in the fifth toe, with a mean of 29.19 ± 2.26 °C.

When comparing plantar foot temperatures between school and sports footwear (Table 1), the Wilcoxon test showed nominally significant (uncorrected) differences in five of the nine ROIs. Heel temperature was nominally higher with sports footwear, at 29.51 ± 1.59 °C compared with 28.68 ± 2.52 °C for school footwear (*p* = 0.0146). Likewise, higher temperatures were recorded with sports footwear in the MLA (*p* = 0.0220), LLA (*p* = 0.0205), central MTHs (*p* = 0.0201), and 5th MTH (*p* = 0.0378). However, these differences did not remain significant after FDR correction (Table 1; q = 0.0535 to 0.0643, i.e., q > 0.05 in all cases).

When comparing dorsal foot temperatures between school and sports footwear (Table 2), the Wilcoxon test showed nominally significant (uncorrected) differences in the medial region, with a mean value of 31.87 ± 1.42 °C for sports footwear compared with 30.92 ± 2.20 °C for school footwear (*p* = 0.0012). The Wilcoxon test also showed nominally significant differences in the lateral region (*p* = 0.0016), 1st MTH (*p* = 0.0286), central MTHs (*p* = 0.0089), and the third toe (*p* = 0.0366). Nevertheless, after FDR correction, only the medial and lateral ROIs remained significant (q = 0.0139 in both cases), with higher temperatures with sports footwear. The adjusted value for the central MTHs (q = 0.0505) lay just above the FDR threshold and was therefore not considered significant.

A significant period effect was found in all ROIs (Supplementary Table S1): in both sequence groups, foot temperature was higher at the second visit than at the first, with estimated period effects of 1.28 to 2.24 °C (Mann–Whitney U = 482.5 to 622.0; exact *p* = 2.3 × 10^−9^ to 3.1 × 10^−7^). In the period-adjusted sensitivity analysis, sports footwear remained associated with higher temperatures, with estimated footwear effects of 0.71 to 1.04 °C. The medial and lateral dorsal regions again showed the strongest evidence (1.03 °C, 95% CI 0.60 to 1.46, and 1.01 °C, 95% CI 0.57 to 1.45, respectively; q = 0.0004 in both cases), and once the period effect was accounted for, the differences in the remaining ROIs also reached significance (q = 0.0036 to 0.0348). The paired differences and their 95% CIs for all ROIs are shown in Supplementary Figure S1.

## 4. Discussion

The results obtained in this study indicate that, on the plantar aspect of the foot (Table 1), regardless of the type of footwear used (school or sports), the MLA exhibited the highest temperatures, whereas the region corresponding to the fifth toe showed the lowest values. Anatomically, the MLA has a richer vascular supply than other plantar regions, which favors greater blood perfusion [27]; this could be related to the higher superficial heat emission detectable by thermography. The higher temperature observed in this region may reflect blood flow from the posterior tibial artery [27]. Consistent with this, numerous infrared thermography studies conducted in adult populations have identified the MLA as the region with the highest mean temperature on the plantar surface, describing it as a characteristic thermal pattern of the healthy foot [28,29,30]. In contrast, the fifth toe region is anatomically more distal and has lower muscle mass and reduced superficial vascularization [31]. We hypothesize that, being further from central circulation and having a reduced functional role in load distribution, this area tends to maintain lower temperatures. Regarding the dorsal aspect of the foot, the lateral region presented the highest temperatures, while the fifth toe region again showed the lowest values (Table 2). From a circulatory perspective, the blood supply of the dorsal foot is primarily provided by the dorsalis pedis artery [32], which converges laterally with branches of the lateral tarsal artery. This confluence may explain the greater blood perfusion in the lateral dorsal region, thereby contributing to its higher temperature [33]. Moreover, the lateral region of the foot experiences greater contact with footwear, which Shimazaki and Murata [9] attribute to the limited airflow that reduces heat loss through convection, which may result in a higher superficial temperature in this area. As observed on the plantar surface, we consider that the reduced peripheral blood flow to the fifth toe, along with its lower muscle volume and limited active mobility, may contribute to the lower temperatures recorded in this region. This thermal pattern is consistent with previous studies that have described temperature gradients from more central and muscular areas towards distal regions that are less vascularized and functionally less active within the foot [22,27,33].

When comparing the two types of footwear, sports footwear was associated with higher mean plantar and dorsal foot temperatures than school footwear in all ROIs (Table 1 and Table 2). On the plantar surface, the Wilcoxon test showed nominally higher temperatures with sports footwear in the heel, MLA, LLA, central MTHs and 5th MTH, with increases of 0.66 to 0.94 °C. However, none of these differences remained statistically significant after FDR correction (q = 0.0535 to 0.0643, Table 1). On the dorsal aspect, the initial comparisons showed higher temperatures in the medial and lateral regions, 1st MTH, central MTHs and third toe (increases of 0.84 to 0.95 °C), but only the medial and lateral regions remained significant after FDR correction (q = 0.0139 in both cases, Table 2). In these two regions, temperatures were 0.95 and 0.94 °C higher with sports footwear, and they showed the largest effect sizes of the study (Rosenthal’s r = 0.311 and 0.303, respectively). Therefore, the most robust footwear-related differences were located on the dorsal aspect of the foot. The other eight nominally significant regions (heel, MLA, LLA, central MTHs and 5th MTH on the plantar surface, and 1st MTH, central MTHs and third toe on the dorsal surface) did not withstand the correction for multiple comparisons, and their effect sizes were small (r = 0.200 to 0.252), whereas those of the remaining seven regions were smaller still (r = 0.132 to 0.184). Nevertheless, the consistent direction of the results, with higher mean temperatures with sports footwear in every ROI, suggests that these differences in the initial test, although slight, could be indicative of a result that should be explored further in future studies. This interpretation is supported by the period-adjusted sensitivity analysis, in which the footwear effect remained of a similar magnitude (0.71 to 1.04 °C) and reached significance in all ROIs once the variance attributable to the period effect had been accounted for. The marked period effect, with higher temperatures at the second visit regardless of footwear, was larger than the footwear effect and may reflect day-specific factors, such as ambient conditions, which can influence skin temperature [8] and which in the present sample were warmer and less humid at the second visit (Section 2.2.2), or the activity performed on each morning. Because the two sequence groups were of similar size, this effect had little influence on the overall footwear estimates, although it increased their variability.

The structural or material mechanisms underlying this association were not directly measured in the present study; nonetheless, several characteristics of sports footwear could plausibly contribute to greater heat retention. Sports footwear typically features thicker soles with varied designs and materials such as synthetic foams (e.g., EVA), which may promote thermal insulation and could contribute to the higher plantar temperatures observed with sports footwear. This is consistent with the mathematical model proposed by Nemati et al. [34], which suggested that heat transfer in footwear depends on the thermal and mechanical properties of the sole. We speculate that cushioned structures incorporated in some models, such as insoles or air chambers, might also reduce convective heat dissipation around the foot. It should be noted, however, that the models of the sports footwear used in this study featured a mesh upper, which could theoretically enhance rather than reduce ventilation and may partially offset heat-retaining effects attributable to other structural components. In contrast, school footwear, owing to its simpler construction and the use of natural leather, a material generally considered to provide good breathability [5], may facilitate greater thermal exchange with the surrounding environment. Overall, the observed difference in foot temperature between footwear types likely reflects a combination of thermal properties inherent to each design, the relative contributions of which remain to be quantified.

Another factor that may contribute to the higher temperatures observed with sports footwear in the medial and lateral dorsal regions, which were the regions with the largest effects in both the primary and the period-adjusted analyses, is fit. According to the footwear characteristics defined in Section 2.2, the sports footwear assessed had a synthetic upper with reinforcements and a thicker cushioned sole, whereas the school footwear had a simpler leather construction; in our view, this may result in a less restrictive fit of school footwear, allowing greater heat dissipation. As both types of footwear used similar fastening systems (Velcro^®^, laces or buckles; Section 2.2), the closure itself is unlikely to explain the differences. Instead, the reinforced synthetic upper and the thicker, multi-layered tongues that, in our experience, are typical of sports shoes, may reduce the space between the dorsal surface of the foot and the shoe upper, potentially limiting ventilation and sweat evaporation, key factors in maintaining lower temperatures on the dorsal aspect of the foot [9]. We hypothesize that this closer fit of the upper over the medial and lateral aspects of the dorsum could explain why these regions were the areas where the footwear effect was most evident. We also speculate that the greater space within the toe box, designed to allow free movement of the toes during activity, may facilitate airflow, helping to maintain a cooler environment in this region.

Regarding the observed differences, both types of footwear maintained mean foot temperature within the range considered to provide thermal comfort (20.00 to 33.00 °C) [6]. However, the higher dorsal temperatures associated with sports footwear, if sustained over longer periods of use, may have implications for foot health, particularly on the dorsal aspect of the foot, which warrants further investigation. From a clinical perspective, podiatrists frequently observe an association between sustained foot warmth, increased sweating, and moisture-related conditions such as blister formation or dermatomycoses [35]; however, this association was not directly assessed in the present study and should therefore be regarded as a clinical hypothesis rather than an established finding.

This study has some limitations, and the results should be interpreted with caution. First, footwear models were not standardized across participants, and variables such as shoe fit and shoe age were not quantified. Moreover, the order of conditions was not randomized, and a strong period effect was found, with higher foot temperatures at the second visit than at the first in all ROIs, regardless of the footwear worn (1.28 to 2.24 °C; Supplementary Table S1). Nevertheless, once this period effect was taken into account, foot temperature with school footwear remained lower than with sports footwear in all ROIs (period-adjusted differences of 0.71 to 1.04 °C). Even so, in a two-period design the period effect cannot be fully distinguished from differences between the sequence groups, which were not randomized and were measured on different days; a carryover of footwear effects from the first to the second visit is unlikely, as the assessments were performed on separate days, but it cannot be formally excluded. Another limitation relates to the clinical interpretation of the observed temperature differences. Even in the regions with the largest effects, the differences between footwear types (0.95 and 0.94 °C) are modest in absolute terms, and whether they are sufficient to produce perceptible thermal discomfort or to increase the risk of dermatological complications remains uncertain. Also, the absence of a baseline temperature measurement and the temporal sequence of the thermographic acquisitions described in Section 2.2.2 could have affected the comparison of regional thermal patterns, mainly in the dorsal region, where the images were taken later. In addition, the reliability of the temperature values was not assessed in the present sample: the individual values of the three images acquired per surface were not retained in the dataset available for analysis, which contained only their mean, so within-session reliability (intraclass correlation coefficient, standard error of measurement and minimal detectable change) could not be computed, and day-to-day reliability could not be estimated within this design, because footwear changed between visits; the repeatability of the procedure therefore relies on previous evidence on ROI placement [18]. The large number of ROI-specific comparisons should be considered when interpreting the statistical findings. As the minimum detectable effect after correction was dz = 0.37, non-significant regional differences should be interpreted as reflecting limited statistical power rather than demonstrated equivalence. Finally, the data were collected in 2018, and their publication was delayed by the authors’ academic and doctoral-thesis schedule. The footwear was classified according to general constructive characteristics rather than specific commercial models, and these characteristics of leather school shoes and synthetic-mesh sports shoes remain representative of current school uniforms in Spain.

Nevertheless, the study also has some strengths. As in vivo studies of the shoe microclimate have shown [9,10], footwear performance emerges from the interaction between material properties, shoe construction, foot morphology, physiological thermoregulation, movement, loading, and individual adaptation to the footwear. In this context, one of the important contributions of the present study is the evaluation of actual thermal responses in children wearing the shoes, rather than inferring these responses solely from laboratory testing of individual footwear components. Future studies should investigate dynamic thermal responses during controlled physical activity and recovery, directly measure intra-shoe temperature and humidity, and examine how specific footwear characteristics interact with individual physiological responses. Studies comparing material-level laboratory testing with in vivo responses in children would help to determine whether the theoretical thermal performance of footwear materials is maintained in real use.

## 5. Conclusions

The pediatric foot exhibits a stable thermal pattern, irrespective of the type of footwear used. The sports footwear used in this sample was associated with higher temperatures on the dorsal aspect of the foot (medial and lateral regions), an association that persisted after accounting for a strong period effect, which was larger than the footwear effect itself. However, the specific footwear characteristics responsible for these differences were not directly assessed. Whether these modest temperature differences have implications for thermal comfort, moisture accumulation, or skin health requires prospective investigation. These findings highlight the importance of considering thermoregulatory properties in the selection of children’s footwear.

## Figures and Tables

**Table 1:** Plantar temperature (°C) of the right foot in each region of interest (ROI) with school and sports footwear (n = 108).

ROIs	Footwear	Mean ± SD (°C)	Difference (95% CI) (°C) ^1^	Median (°C)	IQR (P25–P75)	*p* ^2^	*r* ^3^	*q* ^4^
Heel	School	28.68 ± 2.52	0.83 (0.25 to 1.40)	29.15	27.05 to 30.70	0.0146	0.235	0.0535
	Sports	29.51 ± 1.59		29.50	28.33 to 30.78			
MLA	School	30.64 ± 2.11	0.66 (0.19 to 1.13)	31.15	29.60 to 32.18	0.0220	0.220	0.0535
	Sports	31.30 ± 1.27		31.50	30.50 to 32.30			
LLA	School	29.61 ± 2.27	0.70 (0.18 to 1.22)	29.90	28.70 to 31.30	0.0205	0.223	0.0535
	Sports	30.31 ± 1.45		30.30	29.33 to 31.48			
1st MTH	School	29.09 ± 2.74	0.73 (0.11 to 1.35)	29.60	27.68 to 31.18	0.0739	0.172	0.0897
	Sports	29.82 ± 1.75		30.00	28.63 to 31.20			
Central MTHs	School	29.61 ± 2.79	0.94 (0.30 to 1.57)	30.40	28.43 to 31.80	0.0201	0.224	0.0535
	Sports	30.55 ± 1.80		30.95	29.33 to 31.98			
5th MTH	School	28.71 ± 2.69	0.79 (0.16 to 1.42)	29.25	27.43 to 30.98	0.0378	0.200	0.0643
	Sports	29.50 ± 1.83		29.75	28.20 to 30.90			
First toe	School	28.56 ± 3.22	0.65 (−0.09 to 1.40)	29.20	27.05 to 31.10	0.1695	0.132	0.1695
	Sports	29.22 ± 2.19		29.60	27.80 to 31.00			
Third toe	School	28.73 ± 3.21	0.85 (0.09 to 1.61)	29.40	26.83 to 31.10	0.0639	0.178	0.0855
	Sports	29.58 ± 2.36		30.00	28.40 to 31.30			
Fifth toe	School	27.86 ± 2.90	0.61 (−0.07 to 1.28)	28.45	26.23 to 29.80	0.1055	0.156	0.1175
	Sports	28.47 ± 2.03		28.60	27.13 to 30.08			

SD, Standard deviation; CI, confidence interval; IQR, Interquartile Range. ^1^ Mean paired difference (sports − school footwear) and its 95% confidence interval (unadjusted, parametric and descriptive). ^2^ Wilcoxon Signed-Rank Test for Related Samples. ^3^ Effect Size (Rosenthal’s r). ^4^ q, *p*-value adjusted for multiple comparisons (Benjamini–Hochberg false discovery rate).

**Table 2:** Dorsal temperature (°C) of the right foot in each region of interest (ROI) with school and sports footwear (n = 108).

ROIs	Footwear	Mean ± SD (°C)	Difference (95% CI) (°C) ^1^	Median (°C)	IQR (P25–P75)	*p* ^2^	*r* ^3^	*q* ^4^
Medial	School	30.92 ± 2.20	0.95 (0.46 to 1.44)	31.45	29.73 to 32.58	0.0012	0.311	0.0139
	Sports	31.87 ± 1.42		32.05	30.90 to 32.90			
Lateral	School	31.15 ± 2.25	0.94 (0.44 to 1.43)	31.65	30.23 to 32.78	0.0016	0.303	0.0139
	Sports	32.09 ± 1.39		32.25	31.30 to 33.00			
1st MTH	School	29.92 ± 2.74	0.84 (0.21 to 1.47)	30.65	28.38 to 32.00	0.0286	0.211	0.0609
	Sports	30.76 ± 1.89		30.90	29.50 to 32.30			
Central MTHs	School	30.71 ± 2.72	0.95 (0.35 to 1.55)	31.35	29.63 to 32.68	0.0089	0.252	0.0505
	Sports	31.65 ± 1.72		31.80	30.70 to 32.90			
5th MTH	School	29.80 ± 2.69	0.73 (0.12 to 1.34)	30.30	28.80 to 31.88	0.0553	0.184	0.0855
	Sports	30.53 ± 1.82		30.65	29.40 to 31.98			
First toe	School	28.90 ± 3.08	0.81 (0.10 to 1.52)	29.70	27.10 to 31.30	0.0654	0.177	0.0855
	Sports	29.71 ± 2.23		29.95	28.23 to 31.30			
Third toe	School	29.19 ± 3.06	0.92 (0.21 to 1.63)	29.95	27.63 to 31.60	0.0366	0.201	0.0643
	Sports	30.11 ± 2.19		30.40	28.83 to 31.78			
Fifth toe	School	28.51 ± 2.95	0.68 (−0.02 to 1.39)	29.05	26.73 to 30.98	0.1106	0.154	0.1175
	Sports	29.19 ± 2.26		29.25	27.80 to 30.68			

SD, Standard deviation; CI, confidence interval; IQR, Interquartile Range. ^1^ Mean paired difference (sports − school footwear) and its 95% confidence interval (unadjusted, parametric and descriptive). ^2^ Wilcoxon Signed-Rank Test for Related Samples. ^3^ Effect Size (Rosenthal’s r). ^4^ q, *p*-value adjusted for multiple comparisons (Benjamini–Hochberg false discovery rate).

## Data Availability

The original contributions presented in this study are included in this article. Further inquiries can be directed to the corresponding author.

## Supplementary Materials

The following supporting information can be downloaded at: https://www.mdpi.com/article/10.3390/children13101436/s1, Table S1: Period (order) effect and period-adjusted footwear effect in the two-period crossover analysis (sports footwear first, n = 57; school footwear first, n = 51); Figure S1. Mean paired differences in foot temperature (sports − school footwear) with their 95% confidence intervals for each region of interest (n = 108). Blue: regions that remained significant after Benjamini–Hochberg false discovery rate correction (medial and lateral dorsal regions; q = 0.0139); grey: regions not significant after correction. The vertical line indicates no difference. Confidence intervals are parametric, unadjusted and descriptive.
