# Supplementary material for: Comparative Analysis of Foot Temperature Associated with the Use of Sports and School Footwear in Children Aged 9 and 10 Years
Source: Children (Basel). 2026 Oct 19;13(10):1436. doi: 10.3390/children13101436 (PMC13703068; doi:10.3390/children13101436)
Supplement: Supplementary file 1 [file children-13-01436-s001.zip › children-4588704-supplementary.pdf]

Supplementary Material

Table S1. Period (order) effect and period-adjusted footwear effect in the two-period crossover analysis (sports footwear first, n = 57; school footwear first, n = 51).

ROI	Period effect (95% CI) (°C)	*U*	*p*[1]	*q*[2]	Footwear effect adjusted for period (95% CI) (°C)	*U*	*p*[3]	*q*[2]
Plantar surface								
Heel	1.54 (1.04 to 2.04)	599.0	1.4×10^{-7}	1.8×10^{-7}	0.91 (0.41 to 1.41)	917.5	0.0010	0.0038
MLA	1.35 (0.95 to 1.75)	521.0	9.5×10^{-9}	2.3×10^{-8}	0.73 (0.33 to 1.13)	943.5	0.0017	0.0041
LLA	1.49 (1.04 to 1.93)	536.0	1.6×10^{-8}	2.8×10^{-8}	0.78 (0.34 to 1.23)	932.0	0.0013	0.0038
1st MTH	1.79 (1.27 to 2.32)	517.5	8.4×10^{-9}	2.3×10^{-8}	0.83 (0.31 to 1.36)	1030.0	0.0091	0.0120
Central MTHs	1.81 (1.27 to 2.36)	536.5	1.7×10^{-8}	2.8×10^{-8}	1.04 (0.49 to 1.58)	932.0	0.0013	0.0038
5th MTH	1.80 (1.26 to 2.33)	525.0	1.1×10^{-8}	2.3×10^{-8}	0.89 (0.35 to 1.42)	974.5	0.0032	0.0054
First toe	2.24 (1.62 to 2.86)	482.5	2.3×10^{-9}	2.3×10^{-8}	0.78 (0.16 to 1.40)	1110.5	0.0348	0.0348
Third toe	2.00 (1.33 to 2.66)	622.0	3.1×10^{-7}	3.1×10^{-7}	0.96 (0.29 to 1.63)	1056.5	0.0146	0.0177
Fifth toe	1.95 (1.37 to 2.52)	516.5	8.1×10^{-9}	2.3×10^{-8}	0.71 (0.14 to 1.29)	1070.5	0.0184	0.0196
Dorsal surface								
Medial	1.34 (0.91 to 1.77)	561.0	4.0×10^{-8}	5.6×10^{-8}	1.03 (0.60 to 1.46)	773.0	2.8×10^{-5}	0.0004
Lateral	1.28 (0.84 to 1.73)	615.5	2.5×10^{-7}	2.7×10^{-7}	1.01 (0.57 to 1.45)	794.0	4.9×10^{-5}	0.0004
1st MTH	1.75 (1.20 to 2.29)	561.0	4.0×10^{-8}	5.6×10^{-8}	0.94 (0.39 to 1.49)	953.0	0.0021	0.0044
Central MTHs	1.58 (1.05 to 2.11)	605.0	1.8×10^{-7}	2.0×10^{-7}	1.03 (0.50 to 1.57)	898.5	0.0006	0.0036
5th MTH	1.66 (1.13 to 2.19)	577.5	7.0×10^{-8}	9.1×10^{-8}	0.82 (0.29 to 1.36)	1019.5	0.0076	0.0111
First toe	2.08 (1.48 to 2.69)	508.0	5.9×10^{-9}	2.3×10^{-8}	0.93 (0.33 to 1.53)	1021.5	0.0078	0.0111
Third toe	2.03 (1.42 to 2.63)	523.5	1.0×10^{-8}	2.3×10^{-8}	1.03 (0.43 to 1.64)	972.5	0.0031	0.0054
Fifth toe	2.04 (1.44 to 2.64)	511.0	6.6×10^{-9}	2.3×10^{-8}	0.80 (0.20 to 1.40)	1068.5	0.0178	0.0196

Period effect: temperature at the second visit minus the first visit, estimated as half the difference between the mean within-participant differences (sports − school footwear) of the two sequence groups. Footwear effect adjusted for period: half the sum of these means. 95% CIs are parametric and unadjusted.

[1] Mann–Whitney U test comparing the within-participant differences (sports − school footwear) between sequence groups; exact two-sided p values.

[2] q, p value adjusted for multiple comparisons across the 17 ROIs (Benjamini–Hochberg false discovery rate).

[3] Mann–Whitney U test comparing the period differences (second − first visit) between sequence groups; exact two-sided p values.

MLA: medial longitudinal arch; LLA: lateral longitudinal arch; MTH: metatarsal head.

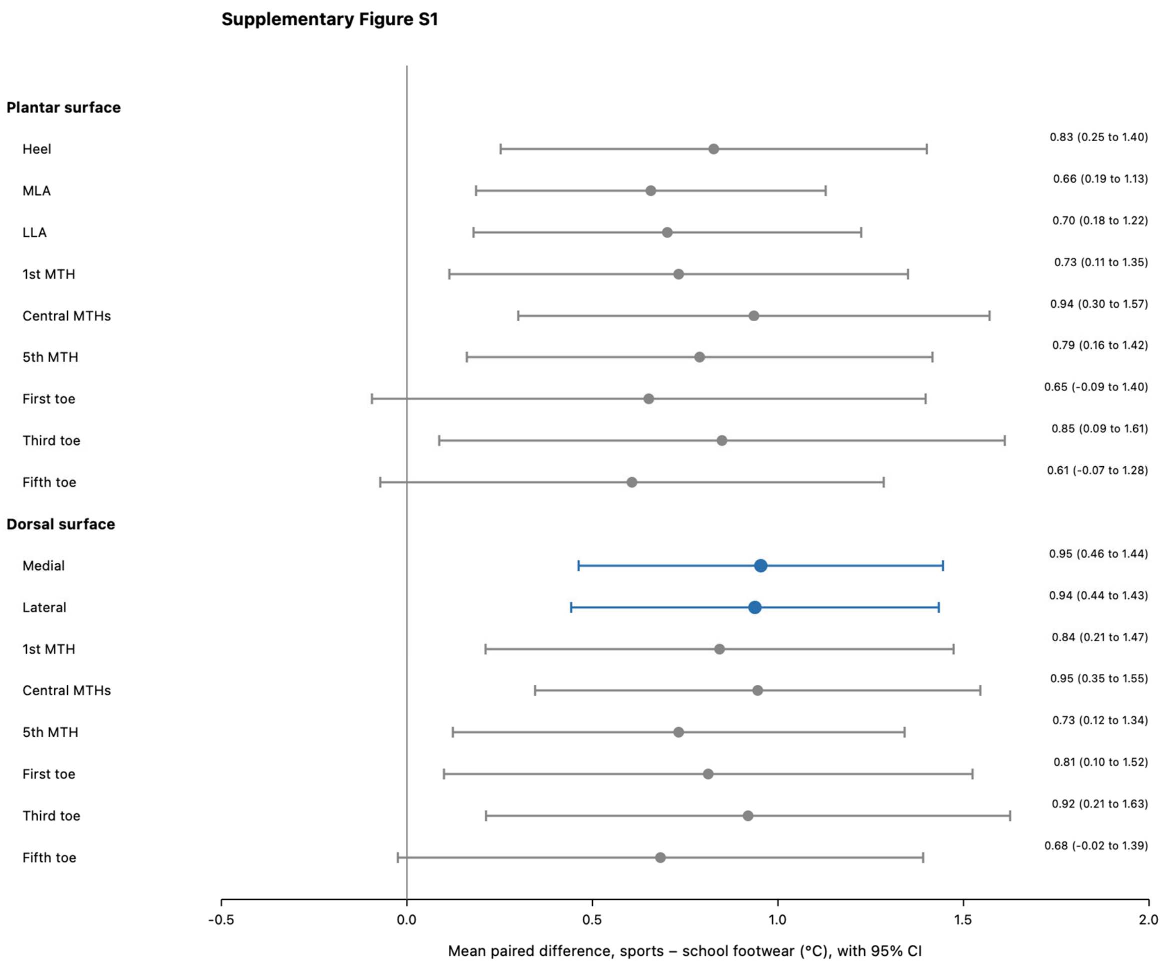

Figure S1. Mean paired differences in foot temperature (sports − school footwear) with their 95% confidence intervals for each region of interest (n = 108). Blue: regions that remained significant after Benjamini–Hochberg false discovery rate correction (medial and lateral dorsal regions; q = 0.0139); grey: regions not significant after correction. The vertical line indicates no difference. Confidence intervals are parametric, unadjusted and descriptive.
